# Exploring epigenetic drift and rare epivariations in amyotrophic lateral sclerosis by epigenome-wide association study

**DOI:** 10.3389/fnagi.2023.1272135

**Published:** 2023-11-27

**Authors:** Alberto Brusati, Silvia Peverelli, Luciano Calzari, Cinzia Tiloca, Valeria Casiraghi, Marta Nice Sorce, Sabrina Invernizzi, Erika Carbone, Rebecca Cavagnola, Federico Verde, Vincenzo Silani, Nicola Ticozzi, Antonia Ratti, Davide Gentilini

**Affiliations:** ^1^Department of Brain and Behavioral Sciences, University of Pavia, Pavia, Italy; ^2^Department of Neurology and Laboratory of Neuroscience, IRCCS Istituto Auxologico Italiano, Milan, Italy; ^3^Bioinformatics and Statistical Genomics Unit, IRCCS Istituto Auxologico Italiano, Milan, Italy; ^4^Department of Medical Biotechnology and Translational Medicine, University of Milan, Milan, Italy; ^5^Department of Endocrine and Metabolic DiseasesI, RCCS Istituto Auxologico Italiano, Milan, Italy; ^6^Department of Pathophysiology and Transplantation, “Dino Ferrari” Center, University of Milan, Milan, Italy

**Keywords:** ALS, epigenetics, bioinformatics, epivariations, EWAS, epigenetic-drift, SEMs, EML

## Abstract

During the last decades, our knowledge about the genetic architecture of sporadic amyotrophic lateral sclerosis (sALS) has significantly increased. However, besides the recognized genetic risk factors, also the environment is supposed to have a role in disease pathogenesis. Epigenetic modifications reflect the results of the interaction between environmental factors and genes and may play a role in the development and progression of ALS. A recent epigenome-wide association study (EWAS) in blood identified differentially methylated positions mapping to 42 genes involved in cholesterol biosynthesis and immune-related pathways. Here we performed a genome-wide DNA methylation analysis in the blood of an Italian cohort of 61 sALS patients and 61 healthy controls. Initially, a conventional genome-wide association analysis was performed, and results were subsequently integrated with the findings from the previous EWAS using a meta-analytical approach. To delve deeper into the significant outcomes, over-representation analysis (ORA) was employed. Moreover, the epigenetic signature obtained from the meta-analysis was examined to determine potential associations with chemical compounds, utilizing the Toxicogenomic Database. Expanding the scope of the epigenetic analysis, we explored both epigenetic drift and rare epivariations. Notably, we observed an elevated epigenetic drift in sALS patients compared to controls, both at a global and single gene level. Interestingly, epigenetic drift at a single gene level revealed an enrichment of genes related to the neurotrophin signaling pathway. Moreover, for the first time, we identified rare epivariations exclusively enriched in sALS cases associated with 153 genes, 88 of whom with a strong expression in cerebral areas. Overall, our study reinforces the evidence that epigenetics may contribute to the pathogenesis of ALS and that epigenetic drift may be a useful diagnostic marker. Moreover, this study suggests the potential role of epivariations in ALS.

## Introduction

Amyotrophic lateral sclerosis (ALS) is a fatal neurodegenerative disorder that affects upper and/or lower motor neurons in the brain and spinal cord, leading to progressive muscle paralysis and death within 2–3 years after the onset. ALS is mainly sporadic (sALS; 90%) with a multifactorial etiology, while familial (fALS) forms are characterized by high genetic heterogeneity. Genetic factors play a key role in ALS etiopathogenesis, although only 75% of fALS and 15% of sALS cases are currently explained by a pool of ~30 causative genes (Akçimen et al., 2023). In this context, whole-genome sequencing (WGS) analyses and genome-wide association studies (GWAS) have widely recognized several genetic risk factors associated with sALS (van Rheenen et al., 2021). However, environmental and lifestyle factors may also contribute as additional and potential risk factors (Ingre et al., 2015). Epigenetic changes, being the results of the interaction between environmental factors and genes, may contribute to clinical presentations and to increased susceptibility of sALS cases (Bennett et al., 2019).

At the state of the art, DNA methylation at CpG sites is the most described epigenetic modification, being the main process that regulates gene expression recruiting gene repressors or, alternatively, inhibiting the binding of transcriptional factors. In addition, DNA methylation may play an important role in genomic stability and imprinting (Benayoun et al., 2015; Elhamamsy, 2017). With the development and refinement of genome-wide arrays aimed at quantifying DNA methylation levels, the number of studies designed to analyze methylome differences between cases and controls has widely increased (Flanagan, 2015). For this purpose, epigenome-wide association studies (EWAS) in blood resulted appropriate for detecting the effects of epigenetic changes potentially induced by several risk factors, including smoking, alcohol intake, body mass index (BMI), and level of inflammatory enzymes, in various neurodegenerative diseases (Marabita et al., 2017; Celarain and Tomas-Roig, 2020). Alterations of epigenetic signatures in ALS disease gained particular interest in the last few years as a valuable and challenging analytical perspective. More specifically, a recent study on a large European cohort provided a list of differentially methylated positions (DMPs) associated with ALS phenotype, demonstrating a possible enrichment for pathways and traits related to metabolism, cholesterol biosynthesis, and immunity (Hop et al., 2022). This study also found that DNA methylation levels at several DMPs and blood cell proportion estimates derived from DNA methylation data were associated with survival rate in patients, suggesting that they might represent indicators of underlying disease processes potentially amenable to therapeutic interventions. However, this study mainly focused attention on DMPs between groups of patients and controls, not considering other epigenetic patterns, such as epigenetic aging acceleration, epigenetic drifts, and the role of rare epivariations. Recently, epigenetic drift, which reflects the accumulation of stochastic epigenetic mutations (SEMs), was reported to be significantly associated with Parkinson’s disease (PD) both at genome-wide level and in PD-causative genes, suggesting that a dysregulated methylome may contribute to the onset of neurodegenerative disorders (Chen et al., 2022). The study also suggests that overall dysregulation of methylation levels, rather than changes in methylation levels at specific loci, are important drivers for outcomes such as aging, pre-term birth, or cancer. This dysregulation of methylation could be a significant factor in the development and progression of PD (Chen et al., 2022). Herein, we investigated the DNA-methylation differences of an Italian cohort of ALS cases and controls by performing an unbiased EWAS in peripheral blood to assess, for the first time, epigenetic drift, both globally and at a single gene level, and the relevance of regions enriched in SEMs, also known as epivariations, in ALS disease.

## Materials and methods

### ALS cohort selection

A cohort of 61 Italian sporadic ALS patients, according to the El Escorial revised criteria (Brooks et al., 2000), were enrolled by IRCCS Istituto Auxologico Italiano. No mutations in the genes listed on the ALS diagnostic panel were found in any of the subjects participating in the study. The panel includes the following genes: *ALS2*, *ANG*, *DCTN1*, *C9ORF72*, *CHMP2B*, *FUS*, *GRN*, *HNRNPA1*, *MATR3*, *NEK1*, *OPTN*, *PFN1*, *SETX*, *SOD1*, *SPAST*, *SPG11*, *HNRNPA2B1*, *MAPT*, *TBK1*, *TUBA4A*, *VAPB*, *SQSTM1*, *TARDBP*, *UBQLN2*, and *VCP*. Informed consent for employing pseudo-anonymized clinical data for research purposes was obtained from all participants (Research ethics board - REB approval, 2021_05_18). Declaration of Helsinki’s guiding principles were followed in conducting the study.

### DNA extraction and EWAs

Genomic DNA (gDNA) was extracted from peripheral blood using the Wizard-genomic DNA purification kit (Promega). Quality control (QC) and quantification were confirmed by visualization of gDNA on 1% agarose gel electrophoresis and NanoPhotometer Pearl (Implen GmbH). Bisulfite conversion was obtained using the EZ DNA Methylation Kit (Zymo Research Corporation). Following the manufacturer’s instructions, NanoPhotometer Pearl was used for assessing the conversion efficiency and the bisulfite DNA (bsDNA) integrity. Samples were analyzed using the Illumina HumanMethylation450 array following the manufacturer’s best practices and the Illumina-supplied reagents and conditions.

### Quality control and differential analysis

QC of probes was first estimated via ChAMP package (Tian et al., 2017). A total of 410,093 probes shared between all samples were retained for the subsequent analysis. In particular, the following criteria were adopted to filter out (1) 4,761 probes with a detection *p*-value above 0.01, (2) 250 probes with a beadcount <3 in at least 5% of samples, (3) 3,051 probes not in CpG Start, (4) 57,628 and 11 probes indicated by Zhou et al. (2017), (5) 9,718 probes located on X and Y chromosomes. In addition, signal intensities were normalized via the SWAN normalization method provided by minfi package (Aryee et al., 2014). Batch effect due to experimental variability was previously evaluated and adjusted with ComBat R methods (Johnson et al., 2007) using the *batch group* (i.e., different groups of experiments) as a covariate. Group-level differential methylation analysis was conducted using the R package limma (Ritchie et al., 2015). Since gDNA samples originated from blood, we considered cellular heterogeneity using the method already described (Houseman et al., 2012). In this context, we used principal component analysis (PCA) to effectively remove multicollinearity between features (cellular components and age).

### Meta-analysis

The tool METAL, a software specifically designed for genome-wide and epigenome-wide data, was used to perform meta-analysis (Willer et al., 2010). This allowed us to combine both site- and genomic region-specific *p*-values obtained from differential methylation analyses. More specifically, we evaluated and retained a total of 382,371 probes from different studies.

### Over-representation analysis, comparative toxicogenomic analysis, and gene prioritization

Over-representation analysis (ORA) was conducted using the online resource ShinyGo (Ge et al., 2020). Comparative Toxicogenomics Analysis was conducted using the online tool provided by Comparative Toxicogenomics Database which combines chemical and genomic data (Davis et al., 2023). The online tool VarElect (Stelzer et al., 2016) was used to prioritize the genes, with the “neurodegeneration” term as the only search parameter.

### Age acceleration estimation

DNA Methylation Age Calculator^1^ was employed for estimating epigenetic aging measures (Horvath, 2013). DNA methylation beta-values were normalized and processed by the DNAmAge calculator^2^ after a further step of normalization computed by the algorithm to make data comparable to the training set. Being data originated from blood, blood cell abundance measures were also calculated, as well as GrimAge, a measure exclusively conceived for blood methylation data. Differences between ALS and controls were finally assessed through non-parametric tests.

### Stochastic epigenetic mutations

We evaluated the presence of stochastic epigenetic mutations (SEMs) as binary scores for each subject-probe datapoint. The determination of SEM status involved computing the interquartile range (IQR) using data from all control samples at each specific locus. Consistent with the predefined criteria, a SEM was considered present for an individual at a given CpG site if its methylation level exceeded three times the IQR below the 25th percentile (Q1–3 × IQR) or three times the IQR above the 75th percentile (Q3 + 3 × IQR) (Gentilini et al., 2015, 2017, 2018; Spada et al., 2020).

Based on the SEM scores obtained at each locus, we evaluated epigenetic drift by calculating 2 Epi Mutation Load (EML) scores for each subject to assess the overall burden of SEM counts across the entire genome (Global-EML) and at a single gene level (Gene-EML).

The association between Global-EML and ALS was tested using a logistic regression model and the same set of covariates used in the EWAS step; the burden of SEMs was expressed on a logarithmic scale and compared between cases and controls.

For testing associations between ALS and Gene-EML (i.e., Gene-specific epigenetic drift scores), we applied methods for rare variants analysis and treated SEM calls at each methylation probe as the variant of interest (Chen et al., 2022). The RVTESTS program, initially used for mapping a contiguous set of rare variants to a specific trait, can be applied to other measures such as copy number variant (CNV), methylation counts, and sequencing data, under the assumption that a cluster of variations in adjacent sites is relevant to the trait. In particular, RVTESTS provides options to adjust for covariates and perform a gene-based Sequence Kernel Association Test (SKAT) that aggregates variants within a gene. By considering the joint effect of multiple rare epigenetic variants, SKAT increases the statistical power to detect associations with rare variant burden, which is often relevant in complex diseases.

### Epivariation analysis

Epivariations are defined as regions exhibiting abnormal methylation patterns and can be identified by their significant enrichment in epimutations (Garg et al., 2020; Gentilini et al., 2023a). These alterations are distinct from epigenetic drift and have been linked to genetic modifications, including CNVs, single nucleotide variations (SNVs), or short tandem repeat (STR) expansions, occurring at the differentially methylated loci. Additionally, inactivating variants in trans-acting factors essential for establishing or maintaining the methylation state at those loci have also been associated with epivariations. We adopted the well-established and validated methodology developed by Gentilini et al. to detect epivariations, wherein we examined genomic regions that exhibited a significant enrichment of SEMs (Gentilini et al., 2018, 2023a,b; Guida et al., 2021). We utilized a sliding window approach with a predefined size on the annotated genome, employing a hypergeometric distribution to assess the significant enrichment of SEMs. This algorithm tests each window by sliding it (one site at a time) and generates a window-associated *p*-value. Through this enrichment, we identified SEMs and repeated the process in adjacent windows to identify SEM-enriched regions. The R package utilized for SEM calculation can be found at DOI 10.5281/zenodo.3813234.

### Statistical analysis and plot generation

Statistical analysis and graphical plots were performed using the R programming language. To analyze differences between cases and controls, the Generalized Linear Regression (glm) model was used taking into account the existing covariates, such as sex, Principal Components, and age. To deal with skewed or non-normal data, values were log converted. In addition, the “Wilcox.test” function offered by the R package “class” was utilized. Images not previously provided by used tools were generated using the “ggplot2” library.

## Results

### Clinical and immunological features of the cohort

A cohort of 61 sporadic sALS cases and 61 controls was enrolled in the study. The ALS population comprised 20 females and 41 males with a median age at the collection date of 50.6 years (Table 1). Age at onset was considered during the selection phase to equally divide the investigated samples into early-onset (<40 years), adult-onset (40–70 years), and late-onset (>70 years; Table 1). Moreover, we selected 36 females and 25 males as controls, with a median age of 48 years. Genome-wide analysis of DNA methylation profiles was performed using the Illumina HumanMethylation450 array. The immunological differences of the analyzed samples in terms of blood cell subpopulations were subsequently estimated. The results of the logistic regression analyses indicate that there was an association between the disease and the unit increase of CD8T, CD4T, NK, Plasmablasts, and Granulocytes (*p*-value < 0.05; Table 1). In contrast, there was no statistically significant association between CD8T naive, CD4T naive, Monocytes, and B-cells and the disease. However, it is important to highlight that a significant relationship was observed between age and gender variables (Table 1).

**Table 1:** Demographic and immunological characteristics of ALS cases and healthy controls.

Demographic Information	ALS cases (*n* = 61)	Controls (*n* = 61)	*p*-value
Gender - frequency (%)			
Female	20 (32%)	36 (59%)	0.006^a^
Male	41 (68%)	25 (41%)	
Age at collection (years) - Median	50.6 (IC 52.6–62.7)	48 (IC 44–51)	0.01^b^
Clinical features			
Age at onset (Years) - Median	48.16	-	-
Early-onset (<40 years)	17	-	-
Adult-onset (40–70 years)	20	-	-
Late-onset (>70 years)	24	-	-
Site of onset			
Spinal	50 (82%)	-	-
Bulbar	11 (18%)	-	-
Inferred immunological cellular components			
	CD8T		0.03^c^
	CD4T		0.03^c^
	CD8 Naive		0.5^c^
	CD4 Naive		0.2^c^
	Natural Killer (NK)		1.19e-05^c^
	Monocytes		0.3^c^
	BCell		0.3^c^
	Granulocytes		0.0001^c^
	PlasmaBlast		0.002^c^

a χ^2^test.

b Wilcoxon test.

c Logistic regression.

### Epigenetic profiling and meta-analysis

**Figure 1:**
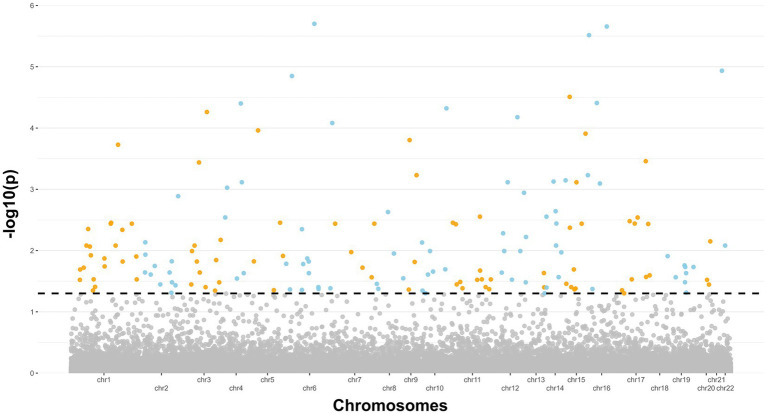
Manhattan plot showing the 27 genes significant emerged from the meta-analysis. The dashed line indicates a significance level of 5% (*p* < 0.05).

After completing methylation data QC and normalization procedures, which included batch effect adjustment, an initial investigation was conducted to examine variations in global DNA methylation profiles between ALS cases and controls. Principal Component Analysis (PCA) was used to reduce the complexity of DNA methylation data, and the results were visualized using a scatterplot featuring the first two principal components (Supplementary Figure 1). Subsequently, a differential analysis was performed, taking into account potential confounding factors such as age and cellular composition. This step retrieved 1,131 significant probes, respectively 1,072 and 59 in hyper and hypo-methylated state (Supplementary Table 1). The findings from this analysis were then combined with those of the previous study conducted by Hop et al. using a meta-analytical approach. Following adjustment for multiple testing, a total of 167 significant and consistent probes corresponding to 126 unique genes (Supplementary Tables 2, 3) were confirmed as differentially methylated and concordant in the state of methylation. Results are also illustrated in the Manhattan plot displayed in Figure 1. Subsequently, an enrichment analysis was conducted on the resulting genes specifically exhibiting hypermethylation and hypomethylation. Regarding the hypermethylated genes, the analysis revealed an enrichment in KEGG pathways such as “Autophagy,” “Longevity regulating pathway,” “Fatty acid metabolism,” “AMPK signaling pathway,” “EGFR tyrosine kinase inhibition resistence,” and “HIF-1 signaling.” Conversely, the down-methylated genes did not reveal any enriched pathway.

We finally explored all chemical compounds potentially associated with the 126 genes identified in the ALS epigenetic signature. To complete this task, we retrieved a list of associated chemical compounds for each gene using the Comparative Toxicogenomic Database. In addition, chemical compounds have been prioritized according to the number of genes they interact with. Intriguingly, this analysis showed an association with (i) compounds already known for their neurotoxic effects, such as sodium arsenite, silicon dioxide, and nickel; (ii) environmental agents such as benzo(a)pirene, air pollution, particulate matter, and tetrachlorodibenzodioxin; (iii) lifestyle factors such as smoking; and (iv) chemical agents used as pesticides such as rotenone, and DDT (Supplementary Figure 2). The prioritized results of this analysis are summarized in Table 2.

**Table 2:** Top 30 chemical compounds associated with resulting differentially methylated genes from the Comparative Toxicogenomic Database.

Chemical compounds	Occurrences (*n*)
Benzo(a)pyrene	250
Valproic Acid	227
Bisphenol A	209
Estradiol	179
Smoke	165
Aflatoxin B1	134
Sodium arsenite	124
Dorsomorphin	107
4-(5-benzo(1,3)dioxol-5-yl-4-pyridin-2-yl-1H-imidazol-2-yl)benzamide	102
Cisplatin	102
Resveratrol	95
Cyclosporine	90
Lipopolysaccharides	88
Fulvestrant	85
Tretinoin	78
Air Pollutants + Particulate	77
Arsenic Trioxide	76
Arsenic	73
Doxorubicin	73
Thallium	72
Dexamethasone	68
Tetrachlorodibenzodioxin	68
Acetaminophen	66
Trichostatin A	61
Glucose	59
Abrine	58
Nickel	57
Aristolochic acid I	56
Cadmium Chloride	54
Jinfukang	53

Chemical compounds are listed according to their occurrence in our dataset.

### Age acceleration

**Figure 2:**
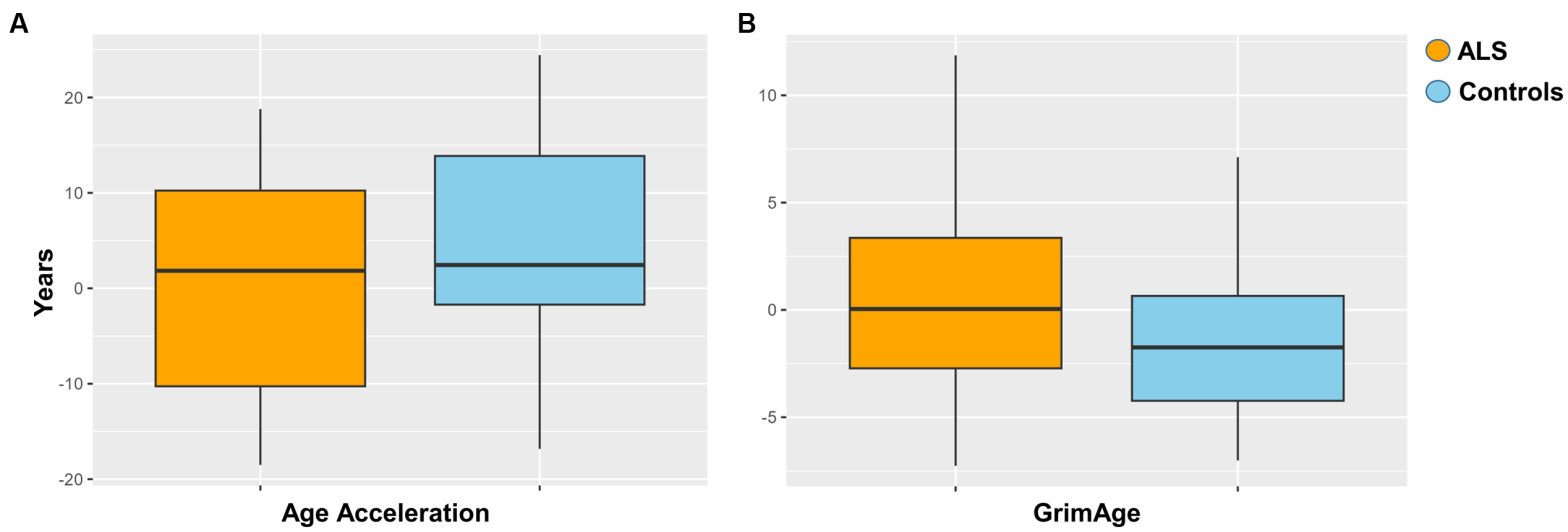
**(A)** Age acceleration between ALS cases and controls (*p* = 0.051). **(B)** Grimage between ALS cases and controls (*p* = 0.4).

Epigenetic age acceleration was estimated via Horvath method for each ALS and control sample as described in Materials and Methods. The resulting regression analysis did not show a statistically significant age acceleration in ALS cases compared to controls (Figure 2A). Additionally, the GrimAge predictor of mortality was assessed, but no statistical evidence between ALS cases and controls was found (Figure 2B).

### Epigenetic drift

We examined epigenetic drift in all samples by considering the burden of SEMs. To assess this burden, we calculated SEM scores at multiple loci and used them to determine two Epi Mutation Load (EML) scores per subject. These two scores, respectively named Global-EML and Gene-EML, allowed us to evaluate the overall accumulation of SEM counts across the entire genome and at specific gene level, respectively. They provided a comprehensive assessment of the SEM burden in each subject.

**Figure 3:**
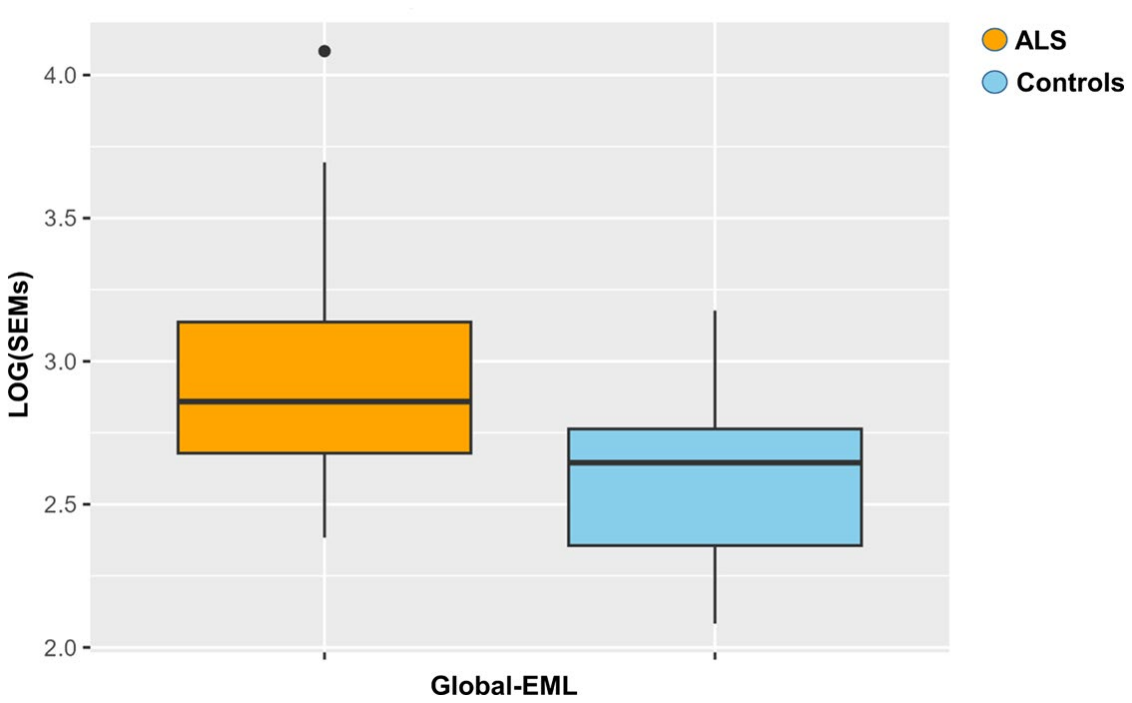
Boxplot showing the epigenetic drift between ALS cases and controls. Differences were assessed by a regression model taking into account several covariates (*p* = 0.0001, β = 0.23).

Global-EML was first log-transformed and subsequently compared between groups by a multiple regression model taking into account important covariates such as gender, age, and cellular composition. This analysis revealed a significantly increased epigenetic drift in ALS cases compared to controls and the risk of ALS was significantly associated with the Global-EML (*p* < 0.0001, β = 0.23; Figure 3).

Moreover, we examined the gene-level epigenetic drift (Gene-EML) using a SKAT test. After adjusting for multiple testing, we observed a distinct set of genes (*n* = 700) that exhibited a significant increase in the accumulation of SEMs among ALS cases compared to controls (Supplementary Table 4). We then conducted an ORA to identify biological pathways potentially affected by the observed epigenetic drift. Notably, this analysis revealed a significant enrichment of pathways associated with “Neurotrophins” with a false discovery rate (FDR) < 0.01 (*n* genes = 12; Table 3). To further expand our analysis, we also investigated the impact of SEMs in the neurotrophin genes and their association with age at the onset of the disease. The multiple regression model, considering also age as a covariate, showed a significant association between the increase in the burden of SEMs and a lower age at the onset (β = −0.43, *p* < 0.01).

**Table 3:** Significant genes enriched in “Neurotrophin signaling pathway” (FDR <0.1) based on SKAT.

Genes	*p*-value	Perm *p*-value
*ABL1*	0.05	0.01
*CAMK4*	0.15	0.01
*KRAS*	0.01	0.002
*MAP2K1*	0.08	0.04
*MAP3K1*	0.01	0.002
*MAPK1*	0.03	0.02
*NFKBIA*	0.03	0.02
*NGF*	0.1	0.01
*PIK3CB*	0.03	0.02
*RAPGEF1*	0.04	0.02
*SH2B1*	0.1	0.01
*SH2B3*	0.02	0.01

### Epivariation analysis

**Figure 4:**
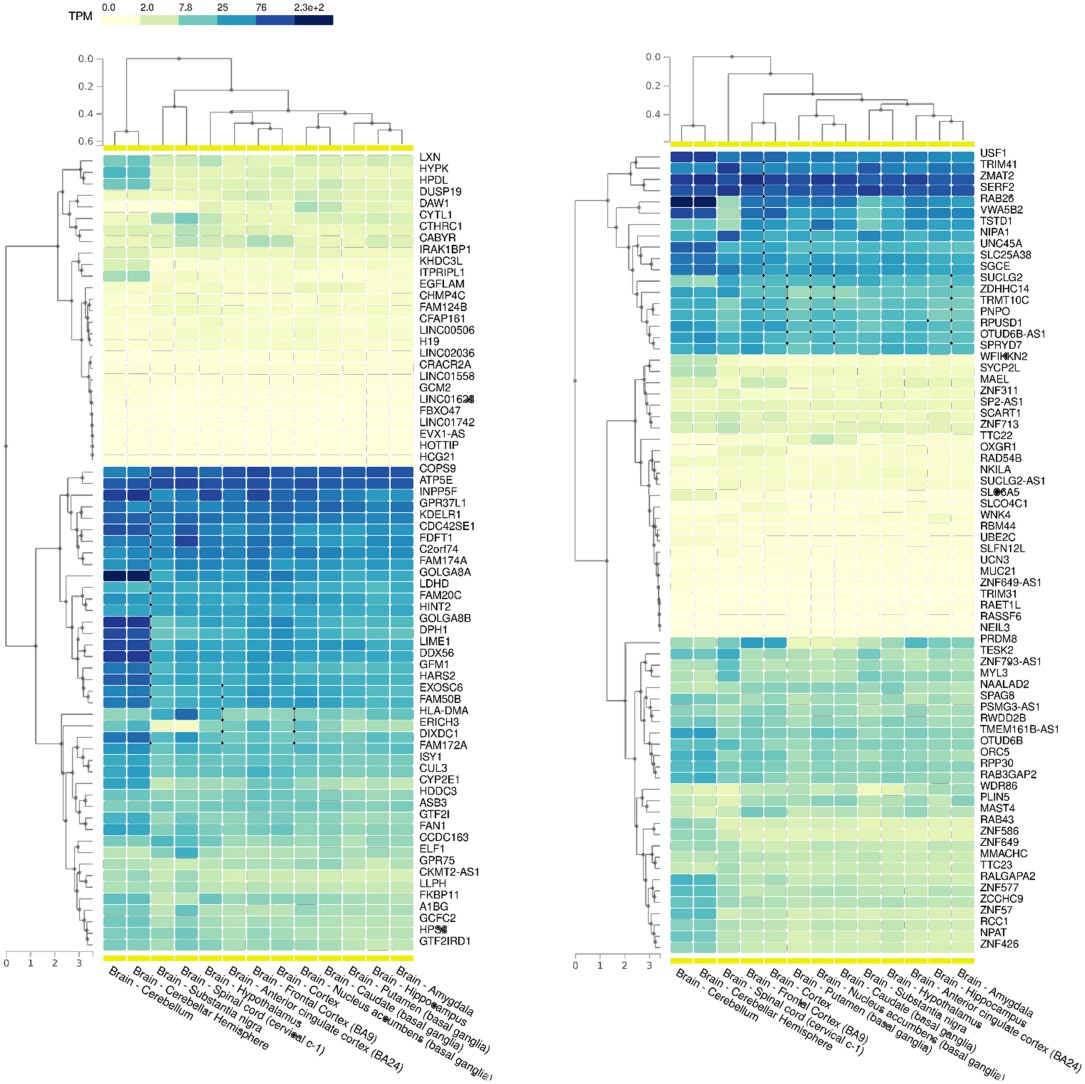
GTEX data showing the expression levels of all the 153 genes with epivariations among the different cerebral areas. Transcript per million (TPM) values were color scaled to display the increased grades of expression.

Based on our SEMs analysis, regions showing a significant enrichment of SEMs, commonly referred to as epivariations, were identified within each subject. While Gene-EML involves comparing all subjects, including both cases and controls, to identify regions with a different burden of epimutations, epivariation analysis focuses on regions within each individual, highlighting the presence of enriched epimutations and aberrant methylation status. The calculation algorithm used is a sliding window approach based on a cumulative hypergeometric test, which conducts an enrichment analysis for each subject (see Materials and Methods). Furthermore, to identify candidate genes, the genomic coordinates of epivariations (SEM-enriched regions) for each individual were recorded along with their corresponding methylation status (hyper- or hypo-methylated), and subsequently annotated (Supplementary Tables 5, 6). From this analysis, a list of genes associated with epivariation, exclusively observed in ALS cases and absent in all controls, was generated (Supplementary Table 7). This investigation revealed 153 unique genes displaying epivariations specific to ALS cases. Among these genes, we further narrowed down the list using GTEX and identified 88 genes that were highly expressed in the brain (Figure 4). Furthermore, the gene prioritization step revealed 2 genes already associated with the term “Neurodegeneration,” namely *NIPA1* and *PRDM8*. *NIPA1* resulted hyper-methylated, while *PRDM8* resulted hypo-methylated. Furthermore, using SNP genotyping data previously obtained in our laboratory (Manini et al., 2023), we also evaluated if CNVs could influence our analysis. No CNVs were detected for all evaluated genes.

## Discussion

The role played by epigenetics in the onset of complex diseases still represents a challenging frontier of study. During the last decade, genome-wide methylation analysis, coupled with an accurate collection of clinical data, has offered extensive knowledge of novel and alternative pathomechanisms that may characterize multifactorial disorders (Horvath, 2013; Gentilini et al., 2015, 2023b; Lu et al., 2020; Spada et al., 2020). In the context of ALS disease, genome-wide approaches and case vs. control studies, although with not completely overlapping results, highlighted several significant genes differentially methylated (Nabais et al., 2020; Cai et al., 2022; Hop et al., 2022; Ruf et al., 2022). In particular, Hop et al. have conducted an accurate and extensive analysis in blood on a large cohort of ALS cases (*n* = 6,763), revealing a clear link between neuroinflammation and disease progression (Hop et al., 2022). The present study, although conducted on a small case–control cohort, extended the previously reported results by including other unexplored epigenetic aspects such as epigenetic drift and rare epivariations.

The differential methylation analysis followed by meta-analysis with previous results (Hop et al., 2022) led to the identification of a more robust episignature. ORA further investigated the epigenetic signature, revealing significant enrichment in specific Gene Ontology categories, in particular in the “Autophagy,” “Longevity regulating pathway,” “Fatty acid metabolism,” “AMPK signaling pathway,” “EGFR tyrosine kinase inhibition resistence” and “HIF-1 signaling” pathways in hypermethylated genes, these results are in line with previous findings (Navone et al., 2015; Hop et al., 2022). Conversely, the down-methylated genes showed no statistical significance in relation to various biological pathways and cellular processes. Furthermore, interesting findings emerged when investigating the chemical compounds likely associated with the identified ALS episignature. The toxicological effects deriving from arsenic derivatives, dioxin, and heavy metals were already well-investigated in many neurological disorders including ALS (Migliore et al., 2015; Prakash et al., 2016; Song et al., 2016; Figueroa-Romero et al., 2020). Similarly, air pollution, pesticides, environmental and/or lifestyle factors were frequently recognized as potential risk factors for neurodegeneration and ALS onset (Zhao et al., 2016; Ash et al., 2017; Marabita et al., 2017; Swash and Eisen, 2020; Malek et al., 2023). Despite the ongoing lack of definitive evidence regarding the clear role of these factors in ALS onset and progression (Ingre et al., 2015; Vasta et al., 2022), the identification of an epigenetic profile compatible with these environmental risk factors provides additional support for the involvement of these chemical compounds in the disease.

Previous studies investigating age acceleration in ALS, frontotemporal dementia (FTD), and PD identified a significant increase in cases compared to controls (Chen et al., 2022; Ruf et al., 2022; Murthy et al., 2023). This result was not confirmed by our study when adjusting for important covariates such as age, gender, and principal components deriving from blood cell type estimates. Similarly, the same negative result was observed even when considering the GrimAge predictor of mortality, which indicates an elevated risk of premature death in the presence of illness.

Interesting findings emerged from our analysis of epigenetic drift, particularly concerning the EML scores, which indicate the accumulation of SEMs at both the genome and gene levels. As recently reported concerning PD, SEM events in blood cells are stochastic in nature, affecting the entire genome and driven by some external process (Chen et al., 2022). However, they seem not only to influence the risk of PD but also motor decline, especially among female patients, as well as time to death in all patients (Chen et al., 2022). Thus, an increased epigenetic drift may have a significant impact on individual health, by likely contributing to increased genomic instability and abnormal gene expression, which can be reflected in some clinical implications. Interestingly, our study showed a significantly increased Global-EML in ALS cases compared to controls, according to the results obtained by the recent literature on PD (Chen et al., 2022). Additionally, the Gene-EML analysis revealed a list of 700 genes exhibiting distinct SEM burdens between ALS cases and controls, leading to a significant enrichment in the “Neurotrophin signaling pathway.” Intriguingly, we also found an association between the burden of SEMs in the resulting 12 neurotrophin genes and the age at the onset. Despite a discouraging history of human trials with neurotrophins in ALS, recent works have hypothesized a role of dysregulated neurotrophin signaling in ALS etiopathogenesis (Baloh et al., 2022; Li et al., 2022). Therefore, our research brought to light a potential epigenetic modification connected to the neurotrophin pathway, a revelation that gains added significance in light of the distinct epigenetic mechanism involving miRNA (Brusati et al., 2022). Altogether, these findings support the possibility that a dysregulated methylome could be associated with ALS (Benayoun et al., 2015). Finally, we focused our analysis on the discovery and significance of uncommon epigenomic changes. Rare epivariations are known to affect hundreds of genes related to several hereditary diseases, suggesting that these genomic modifications may help explain the mutational spectrum underpinning many Mendelian disorders (Garg et al., 2020). Epivariations are regions or genes characterized by aberrant methylation. They are identified as regions with enrichment of SEMs, but they differ from the regions highlighted in the Gene-EML analysis because SEM enrichment is calculated within each individual subject rather than across subjects. Our analysis revealed 153 genes carrying epivariations exclusively in ALS cases, with 88 of them highly expressed in brain and specific cerebral areas. Additionally, the prioritization analysis indicated that a couple of genes were already associated with “Neurodegeneration.” *NIPA1*, which was hypermethylated, has been reported as a common risk factor for ALS, where the expansion of the repetition unit motif GCG > 8 has been linked to an increased risk of the disease (Tazelaar et al., 2019), but associations with its methylation status were not investigated. On the other hand, *PRDM8*, which showed hypomethylation and codes for histone methyltransferase acting as a negative regulator of transcription, has never been linked to ALS, although recent research has shown its involvement in the specification of motor neurons and oligodendrocytes (Scott et al., 2020). A recent study on *PRDM8* knocked-down cells also showed that the loss of *PRDM8* leads to impaired neuronal differentiation (Cypris et al., 2020). This study found that CpGs associated with *PRDM8* were hypomethylated in knocked-down cells compared to control cells, thus supporting our findings in ALS patients.

The primary strength of this study is its rigorous and extensive evaluation of methylation differences using a multilevel approach, which not only enhances our understanding of the epigenetic aspects associated with ALS but also allows for a more in-depth exploration of the intricate and multifaceted mechanisms underlying the disease. Several limitations of our study should be considered when interpreting our results. The relatively small sample size is certainly a restrictive factor that may affect retrieved information, although the meta-analytic approach we conducted increased our statistical power. We are fully aware that a larger sample size may be needed to confirm our findings. Another limitation is the study’s cross-sectional design, which prevented us from inferring causality or temporal relationships between epigenetic modifications and the development of ALS. Longitudinal studies of the same individuals over time would be needed to address this issue. Additionally, our study focused on a single Italian ALS cohort, and findings may not be generalizable to other populations. Further studies will therefore be needed to confirm our findings in populations with different ethnic origins and to determine whether the observed epigenetic modifications are specific to the Italian population or more broadly applicable to individuals with ALS worldwide. Another limiting aspect of our research was certainly the inability to further extrapolate information regarding genetic expression and exposure factors from our case data. We additionally know that associating specific epigenetic variation across different tissues, such as brain and blood, still remains challenging. However, the use of blood as a study tissue for epigenetic investigations into neurodegenerative diseases is supported by several justifications. These include:

Correlation between blood and brain tissues: there is a substantial degree of epigenetic variability correlation between blood and brain tissues, as supported by the findings by Davies et al. (2012) and other relevant publications. Despite variations in epigenetic regulation between these two tissue types, extensive research has consistently revealed epigenetic modifications in genes associated with neurological processes when studying blood as a valuable resource for investigating psychiatric and neurodegenerative disorders.
Accessibility and ease of collecting blood samples: blood is an attractive tissue for study due to its accessibility and ease of collection. This practical advantage positions blood as an invaluable resource for the discovery of potential biomarkers, particularly given the expeditious collection of blood samples relative to other tissue types. This convenience enhances its applicability for clinical and diagnostic purposes.

Finally, our study examined DNA methylation at the whole genome level, but it is also likely that other epigenetic modifications, such as histone modifications or DNA hydroxymethylation, may also play a role in the pathogenesis of ALS. Further analysis will be required to examine these other epigenetic modifications and to determine their potential relevance to the disease.

Overall, our study provides additional evidence that epigenetics may be involved in the pathogenesis of sALS and that epigenetic drift may be a useful marker for the disease. In particular, we found a Global-EML increase in ALS and an association between age at onset and the burden of SEMs in neurotrophin genes by Gene-EML analysis. However, these findings need further confirmation by a deep examination of the mechanisms underlying the observed epigenetic modifications. Moreover, epivariations analysis highlighted two genes already associated with ALS or neural impairment. However, as epivariations are a new analytical approach for DNA methylation analysis, further investigations to evaluate their influence on ALS will be required.

## Data availability statement

The datasets presented in this study can be found in online repositories. The names of the repository/repositories and accession number(s) can be found at: https://www.ncbi.nlm.nih.gov/geo/query/acc.cgi?acc=GSE239901.

## Ethics statement

The studies involving humans were approved by Research ethics board IRCCS Istituto Auxologico Italiano- REB approval, 2021_05_18. The studies were conducted in accordance with the local legislation and institutional requirements. The participants provided their written informed consent to participate in this study.

## Author contributions

AB: Data curation, Formal analysis, Investigation, Methodology, Software, Writing – original draft, Writing – review & editing. SP: Data curation, Investigation, Methodology, Validation, Writing – review & editing. LC: Data curation, Formal analysis, Methodology, Software, Validation, Writing – review & editing. CT: Conceptualization, Data curation, Investigation, Writing – review & editing. VC: Data curation, Writing – review & editing. MS: Data curation, Writing – review & editing. SI: Writing – review & editing. EC: Investigation, Software, Writing – review & editing. FV: Data curation, Writing – review & editing. VS: Resources, Supervision, Writing – review & editing. NT: Funding acquisition, Project administration, Resources, Supervision, Writing – review & editing. AR: Funding acquisition, Project administration, Resources, Supervision, Writing – review & editing. DG: Conceptualization, Formal analysis, Investigation, Methodology, Software, Supervision, Writing – original draft, Writing – review & editing. RC: Software, Methodology, Data curation, Validation.

## Footnotes

^1^ https://horvath.genetics.ucla.edu/html/dnamage/

^2^ https://dnamage.genetics.ucla.edu/home
