# Supplementary figures and images for: Exploring epigenetic drift and rare epivariations in amyotrophic lateral sclerosis by epigenome-wide association study
Source: Front Aging Neurosci. 2023 Nov 27;15:1272135. doi: 10.3389/fnagi.2023.1272135 (PMC10711632; doi:10.3389/fnagi.2023.1272135)

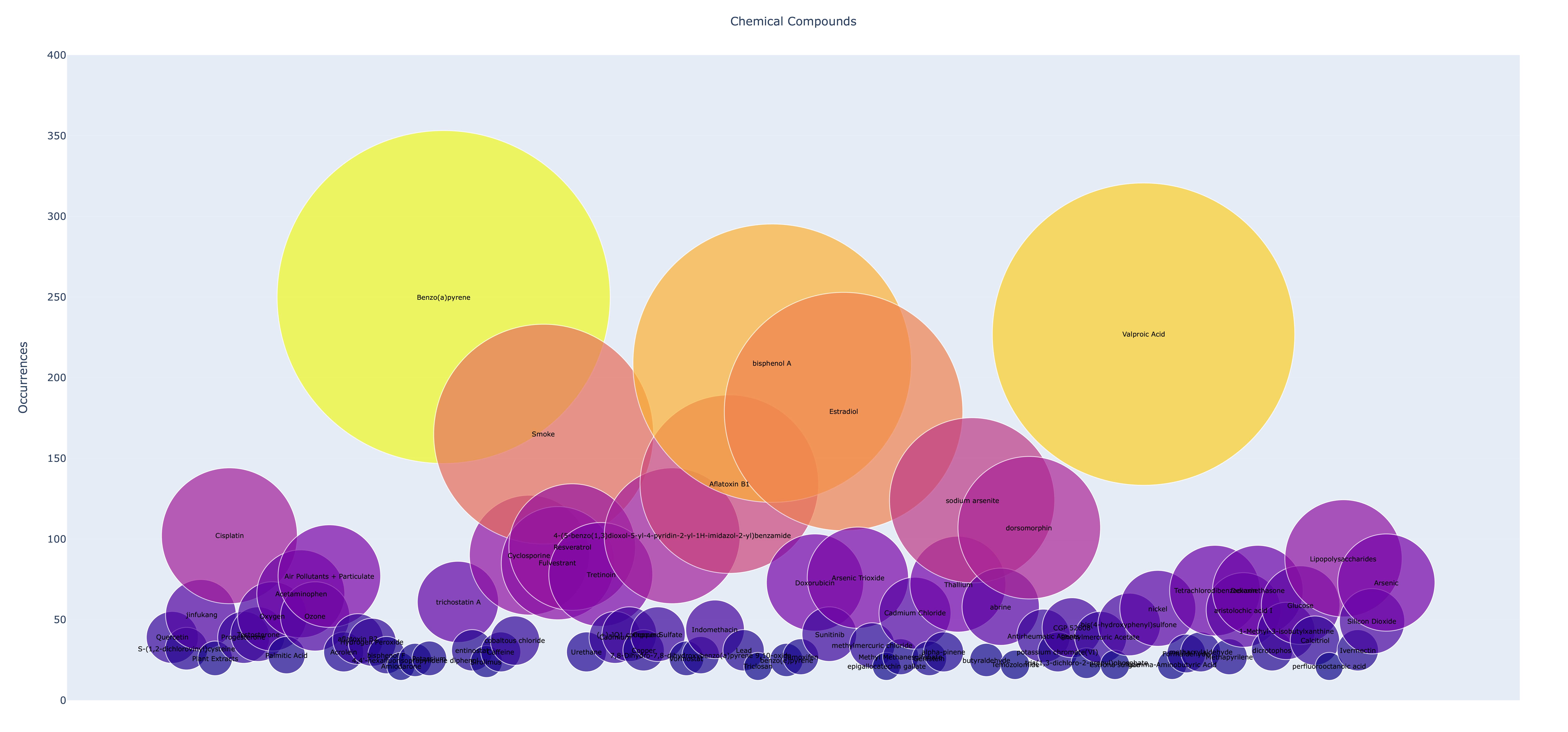

Supplement: Supplementary file 3 [file Image_1.TIF]

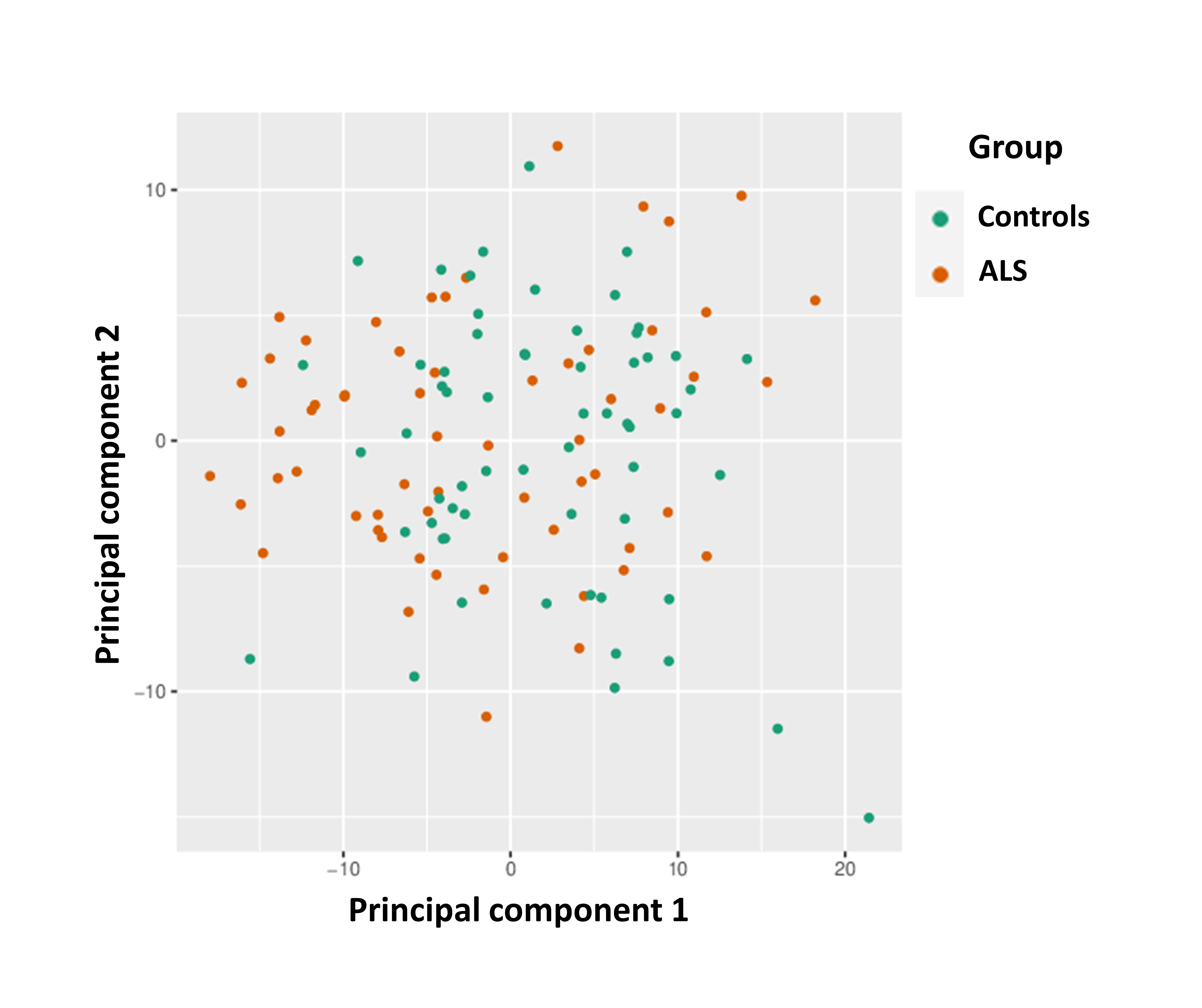

Supplement: Supplementary file 4 [file Image_2.TIF]
